# Emotional attention capture by facial expressions

**DOI:** 10.1038/srep14042

**Published:** 2015-09-14

**Authors:** Reiko Sawada, Wataru Sato

**Affiliations:** 1 Primate Research Institute, Kyoto University, 41-2 Kanrin, Inuyama, Aichi, 484-8506, Japan

## Abstract

Previous studies have shown that emotional facial expressions capture visual attention. However, it has been unclear whether attentional modulation is attributable to their emotional significance or to their visual features. We investigated this issue using a spatial cueing paradigm in which non-predictive cues were peripherally presented before the target was presented in either the same (valid trial) or the opposite (invalid trial) location. The target was an open dot and the cues were photographs of normal emotional facial expressions of anger and happiness, their anti-expressions and neutral expressions. Anti-expressions contained the amount of visual changes equivalent to normal emotional expressions compared with neutral expressions, but they were usually perceived as emotionally neutral. The participants were asked to localize the target as soon as possible. After the cueing task, they evaluated their subjective emotional experiences to the cue stimuli. Compared with anti-expressions, the normal emotional expressions decreased and increased the reaction times (RTs) in the valid and invalid trials, respectively. Shorter RTs in the valid trials and longer RTs in the invalid trials were related to higher subjective arousal ratings. These results suggest that emotional facial expressions accelerate attentional engagement and prolong attentional disengagement due to their emotional significance.

Emotional signals are proposed to be prioritized during resource-limited human information processing because of their adaptive significance^1^. Such evolutionary perspective has been empirically supported by studies utilizing the spatial cueing paradigm^2^,^3^,^4^. In these studies, researchers presented emotional or neutral facial expressions as non-predictive cues in the peripheral visual fields of participants before the presentation of the target at either the same (valid trial) or the opposite (invalid trial) location. In the cueing paradigm, shorter RTs in valid trials and longer RTs in invalid trials indicate the acceleration of initial attentional engagement and the prolongation of attentional disengagement, respectively^5^. The reaction times (RTs) to localize or detect targets were longer when the targets were preceded by invalid emotional expression cues than when they were preceded by invalid neutral expression cues. Therefore the results indicated that emotional facial expressions prolonged the disengagement of attention^2^,^3^,^4^.

However, it remains unclear whether such attentional modulation, which is triggered by emotional faces, is attributable to emotional or to visual factors. Emotional and neutral facial expressions differ not only in emotional significance but also in visual features (e.g., oblique eyebrows in angry faces and horizontal eyebrows in neutral faces). Several studies have demonstrated that certain visual features, such as oblique lines, were more efficiently processed than were other features, such as horizontal lines^6^, suggesting that changes in physical features may play an important role in the attentional modulation by emotional facial expressions.

In the literature of visual search paradigm, some recent studies investigated the effect of emotional versus visual factors on the detection of photographic facial stimuli^7^,^8^. The researchers presented normal emotional facial expressions of anger and happiness or control stimuli, termed “anti-expressions”^9^, within the crowd of neutral expressions. The anti-expressions were created using computer-morphing techniques; they contained visual changes that were equivalent to those in the normal emotional facial expressions compared with neutral expressions^9^, but they were most frequently labeled or categorized as emotionally neutral expressions^8^,^9^. Therefore, it is suggested that the anti-expressions are usable as one of control stimuli for emotional facial expressions in an examination of visual properties^9^. The studies showed that the RTs for detecting normal expressions were shorter than were those for detecting anti-expressions^7^,^8^, indicating that emotional facial expressions are efficiently processed due to emotional rather than to visual factors. Moreover, these findings corroborate the proposal of the neurocognitive mechanism, suggesting that neural mechanisms for visual and emotional attention may be dissociable^10^. Hence, we hypothesized that attentional modulation by emotional facial expressions would be attributable to emotional significance rather than to visual features.

To test this hypothesis, we conducted the cuing task using normal emotional facial expressions of anger and happiness and their anti-expressions as cues (Fig. 1). The cueing task, compared with visual search task, is the more effectual method for testing the attentional effect, because the cueing task allows us to separate initial engagement and disengagement of visual attention, whereas the results of the visual search task reflect both target and distractor effects^11^. We also presented neutral expressions as cues to compare the results with those in previous studies^2^,^3^,^4^. Moreover, we asked participants to rate their subjective emotional arousal and valence^12^ to investigate the relationship between their emotional reaction and attentional modulation. Additionally, we tested the subjective feelings of stimulus familiarity and naturalness as possible confounding factors^13^. We predicted that (1) RTs for target localization under the invalid condition would be longer in response to normal expression cues than to anti-expression cues or to neutral expression cues; and (2) the degree of attention modulation in RTs would be related to ratings of emotional arousal.

## Results

### RT

Mean (with *SE*) RTs are shown in Fig. 2. We performed a repeated-measure analysis of variance (ANOVA) on RTs with type (normal/anti-expression) and emotion (anger/happiness), and validity (valid/invalid) as within-participant factors. The results showed a significant main effect of validity, *F*(1, 33) = 6.96, *p* < .05, *η*_p_^2^ = .17, and an interaction between type and validity, *F*(1, 33) = 8.93, *p* < .01, *η*_*p*_^2^ = .21. Follow-up analyses of the interaction revealed a significant simple-effect of type in valid, *F*(1, 66) = 5.93, *p* < .05, and invalid trials, *F*(1, 66) = 6.78, *p* < .05, indicating that the RTs for targets preceded by normal expressions were shorter in valid trials and longer in invalid trials than those for targets preceded by anti-expressions.

To compare the results with those in previous studies^2^,^3^,^4^, multiple comparisons with Bonferroni correction between normal/anti-expression condition and the neutral expression condition were conducted on RTs for each validity condition. In the invalid trials, the RTs to localize targets were significantly longer for normal expressions of anger and happiness than for neutral expressions, *t*(132) > 4.24, *p*s < .001, *rs* > .35, whereas anti-expressions of both anger and happiness were not different from neutral expressions, *t*s(132) < 2.63, *p*s > .09. In the valid trials, the results for normal and anti-expressions did not differ significantly from those for neutral expressions, *t*s(132) < 0.45, *p*s > .10.

### Rating

Mean (with *SE*) ratings are shown in Table 1. First, we conducted a two-way ANOVA with type (normal/anti-expression) and emotion (anger/happiness). Then, to compare the rating scores of normal/anti-expressions of anger and happiness with neutral expression, multiple comparisons with Bonferroni correction were conducted.

### Arousal

The ANOVA revealed significant main effects of type, *F*(1, 33) = 104.77, *p* < .001, and of emotion, *F*(1, 33) = 6.18, *p* < .05. The interaction was not significant, *F*(1, 33) = 0.04, *p* > .10. The results indicate that normal expressions were higher arousal than anti-expressions and that angry expressions were higher arousal than happy expressions.

Multiple comparisons revealed that normal expressions of anger and happiness were higher arousal than neutral expressions, *t*s(132) > 10.40, *p*s < .001, *rs* > .67. They showed that anti-happiness were not different from neutral expression, *t*(132) = 1.88 , *p* > .10, whereas anti-anger were slightly higher than neutral expression, *t*(132) = 3.59 , *p* < .05, *r* = .30.

### Valence

The ANOVA revealed a significant main effect of emotion, *F* (1, 33) = 128.44, *p* < .001, and a significant interaction between type and emotion, *F* (1, 33) = 176.29, *p* < .001. Follow-up analyses for the interaction revealed a significant simple-effect of type for anger, *F*(1, 66) = 76.73, *p* < .001, and for happiness, *F*(1, 66) = 111.49, *p* < .001, indicating that normal-anger was more unpleasant than anti-anger and that normal-happiness was more pleasant than anti-happiness. The analyses also revealed a significant simple-effect of emotion for normal expressions, *F* (1, 66) = 212.57, *p* < .001, and for anti-expressions, *F* (1, 66) = 26.41, *p* < .001, indicating that normal-anger was more unpleasant than normal-happiness and that anti-anger was more pleasant than anti-happiness.

Multiple comparisons revealed that normal-anger was more negative, *t*s(132) = −16.56, *p* < .001, *r* = .82, and normal-happiness was more positive, *t*(132) = 7.42, *p* < .001, *r* = .54, than neutral expression. The analyses showed that anti-anger was not different from neutral expression, *t*(132) = −1.40, *p* > .10, whereas anti-happiness was more negative than neutral expression, *t*(132) = −7.08 , *p* < .001, *r* = .52.

### Familiarity

The ANOVA revealed significant main effects of type, *F*(1, 33) = 5.86, *p* < .05, and emotion, *F*(1, 33) = 48.45, *p* < .001, and a significant interaction between type and emotion, *F*(1, 33) = 107.17, *p* < .001. Follow-up analyses revealed a significant simple-effect of type for anger, *F*(1, 66) = 26.85, *p* < .001, and for happiness, *F*(1, 66) = 93.53, *p* < .001, indicating that normal-anger was less familiar than anti-anger and that normal-happiness was more familiar than anti-happiness. The analyses also showed the significant simple-effect of emotion for normal expressions, *F*(1, 66) = 114.50, *p* < .001, and for anti-expressions, *F*(1, 66) = 14.97, *p* < .001, indicating that normal-anger was less familiar than normal-happiness and that anti-anger was more familiar than anti-happiness.

Multiple comparisons revealed that normal-anger, anti-anger and anti-happiness were less familiar than neutral expressions, *t*s(132) < −5.26, *p* < .001, *r* > .42, whereas only normal-happiness was not different from neutral expressions, *t*(132) = 1.86, *p* > .10

### Naturalness

The ANOVA revealed a significant main effects of type, *F*(1, 33) = 6.66, *p* < .05, and emotion, *F*(1, 33) = 35.09, *p* < .001, and a significant interaction between type and emotion, *F*(1, 33) = 25.69, *p* < .001. Follow-up analyses revealed the significant simple-effect of type only for happiness, *F*(1, 66) = 26.51, *p* < .001, indicating that normal-happiness was more natural than anti-happiness. The analyses also showed the significant simple-effect of emotion only for normal expressions, *F*(1, 66) = 42.49, *p* < .001, indicating that normal-anger was less natural than normal-happiness.

Multiple comparisons revealed that normal-anger and anti-expressions of anger and happiness were less natural than neutral expressions, *t*s(132) < −6.24, *p* < .001, *r* > .48, whereas only normal-happiness was not different from neutral expressions, *t*(132) = −0.38, *p* > .10

### Rating-RT relationship

Regression analyses were performed to examine the relationship between emotional reactions and attentional effects by facial cues in each validity condition. The results revealed a significant negative relationship between arousal ratings and RTs in the valid trials, *t*(101) = −1.96, *p* = .05, indicating higher arousal ratings for shorter RTs in valid trials (Fig. 3(a)). We also observed a significant positive relationship between arousal ratings and RTs in the invalid trials, *t*(101) = 2.11, *p* < .05, indicating higher arousal rating for longer RTs in invalid trials (Fig. 3(b)).

No significant relationship was found between valence and RTs, absolute *t*s(101) < 0.92, *p*s > .10, between familiarity and RTs, absolute *t*s(101) < 0.61, *p*s > .10, or between naturalness and RTs, absolute *t*s(101) < 0.95, *p*s > .10.

## Discussion

Our RT results showed that normal expressions of anger and happiness, compared with neutral expressions, delayed responses to subsequent targets under the invalid condition. This result is consistent with those of several previous studies^2^,^3^,^4^, indicating that emotional facial expressions prolong the disengagement of attention compared with neutral ones.

More importantly, our RT results showed that the normal expressions of anger and happiness also delayed responses to subsequent targets compared with their anti-expressions under the invalid condition. Because the anti-expressions reflected a controlled degree of visual changes but categorized most frequently as neutral expressions consistent with previous studies^8^,^9^, the result indicates that, compared with neutral expressions, emotional facial expressions prolong attentional disengagement due to their emotional rather than to their visual factors.

Additionally, our RT results showed that, compared with anti-expressions, normal expressions quickened responses to subsequent targets in the valid condition, indicating the facilitation of attentional engagement. This facilitative effect on attentional engagement has not been evident when normal expressions were compared with neutral expressions in this or previous studies^2^,^3^,^4^. However, our results are consistent with those of a previous study showing the facilitation of attentional engagement by emotional words compared with neutral words^14^. Moreover, the results are consistent with other studies which have reported the facilitation of visual processing by emotional facial expressions using different tasks^15^. Therefore, we suggest one possibility that our control of the visual aspects of normal and anti-expressions might have rendered the facilitative effect of emotion on attentional engagement.

Furthermore, the results of regression analyses revealed the relationship between enhancement of subjective emotional arousal and prolongation of RTs under the invalid condition. The regression analyses also revealed that enhancement of emotional arousal were related to shortening of RTs under the valid condition. Because the arousal ratings reflect the intensity of emotions^12^, these results indicate that facial expressions that induce intense emotions of the perceivers modulate not only difficulty of attentional disengagement but also the facilitation of attentional engagement to facial expression cues. Our results also showed no significant relationship between valence and RTs under either valid or invalid conditions. These results suggest that enhanced subjective emotional arousal is associated with the attentional capture by emotional pictures, consistently with previous study^16^. Moreover, there was no significant relationship between familiarity/naturalness and RTs, suggesting that the effect of the non-emotional processes were not accounting for attentional engagement and disengagement by emotional facial expressions. The data supports our hypothesis that the emotional significance drives attentional modulation by emotional facial expressions.

Taken together, our results indicate that the facial expressions speeded the engagement and prolonged the disengagement of attention due to their emotional significance rather than to their visual features. The results are consistent with those of previous studies using the visual search paradigm that have shown that emotional faces were detected more efficiently because of their emotional significance, not of visual features^7^,^8^. However, such results were suggestive regarding attentional capture, because performances of the visual search task reflected the effects of both targets and distracters^11^. Our results are also consistent with theoretical models including specialized neurocognitive mechanisms for emotional attention that are independent of those for the attention to the visual features^10^. To our knowledge, the current study is the first report to show that emotional signals of facial expressions modulate initial engagement and disengagement of visual attention because of their emotional significance rather than of their visual changes.

Our results have several implications related to neural mechanisms. A previous theoretical study suggested that the emotional attention may be implemented by the enhanced activation of visual cortices, which is associated with the modulatory influence from the amygdala, in response to emotional versus neutral stimuli^10^. An electrophysiological study reported that visual cortices showed greater activation in response to emotional than to neutral expressions, which was attributed to emotional rather than visual factors^8^. A neuroimaging study found that amygdala activation in response to emotional versus neutral facial expressions was associated with emotional rather than visual processing^17^. Together with these data, our results suggest that increased visual attention to emotional facial expressions may be related to the enhanced activation of the visual cortices via the activation of the amygdala.

Although our preliminarily experiment confirmed that anti-expressions were most frequently categorized as neutral expressions consistently with previous studies^8^,^9^, the results of subjective ratings of anti-expressions were not completely comparable with those of neutral expressions, inconsistent with that in the previous study^8^. The methodological differences, such as higher ratio of female of the participants (47.1% vs. 30.0%), may explain the discrepant results. Some studies indicated that individual difference, such as gender and personality, modulate the subjective ratings for the facial expressions^18^,^19^. Further studies controlling the individual differences of participants would allow to represent anti-expressions as neutral expressions and to test more rigidly the emotional versus visual effect on the perception of emotional facial expressions.

A limitation of this study can be acknowledged. We examined only one condition involving a stimulus onset asynchrony (SOA) between the cue and target to simplify the experimental design. However, previous studies have reported that the attentional shifts based on visual features or intentions can change depending on cue-target SOAs^20^, suggesting the possibility of such changes with regard to emotional attention. Promising directions for future research include the elucidation of the time course of emotional attention capture by emotional facial expressions.

## Methods

### Participants

Thirty-four volunteers (16 females, *M* ± *SD* age, 22.9 ± 4.1 years) participated after providing written informed consent for the experimental procedure, which was approved by the Ethics Committee of Primate Research Institute, Kyoto University. The study was also conducted in accord with the Declaration of Helsinki. All participants were right-handed as assessed by the Edinburgh Handedness Inventory^21^ and had normal or corrected-to-normal visual acuity.

### Stimuli

Normal and anti-expressions of anger and happiness and normal neutral expressions were used as cues and an open dot was used as a target. Each individual face subtended a visual angle of 4.8° horizontally ×6.3° vertically. A target dot subtended a visual angle of 3.1° × 3.1°.

**Figure 1:**
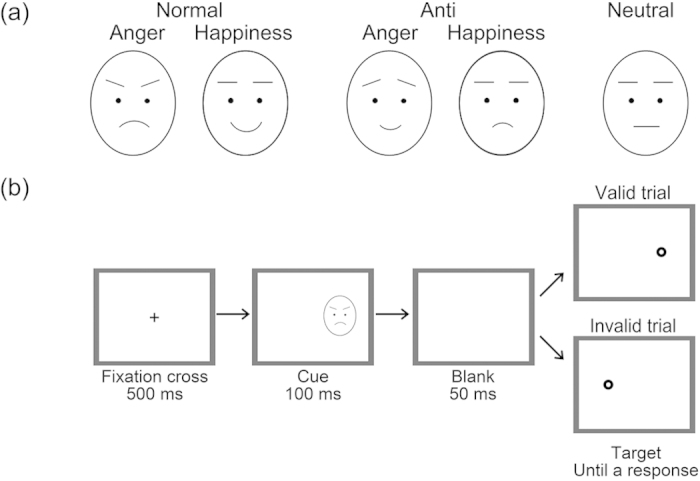
Schematic illustrations of stimuli (a) and presentation of cue and target stimuli in the valid and the invalid trials in the cueing task (b). Actual stimuli were photographic faces (see Fig. 1 in the previous study^7^).

**Figure 2:**
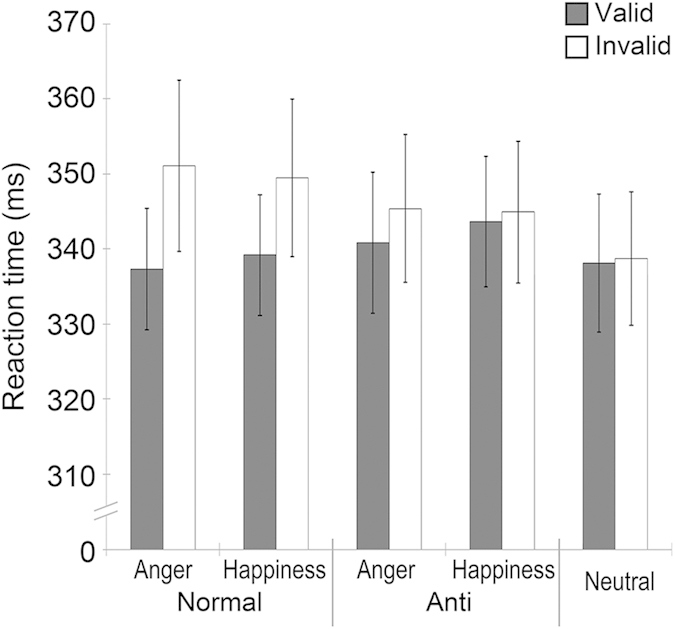
Mean (with *SE*) reaction time (RT) of localizing target stimuli for the cue condition of normal and anti-expressions of anger and happiness and neutral expressions.

**Figure 3:**
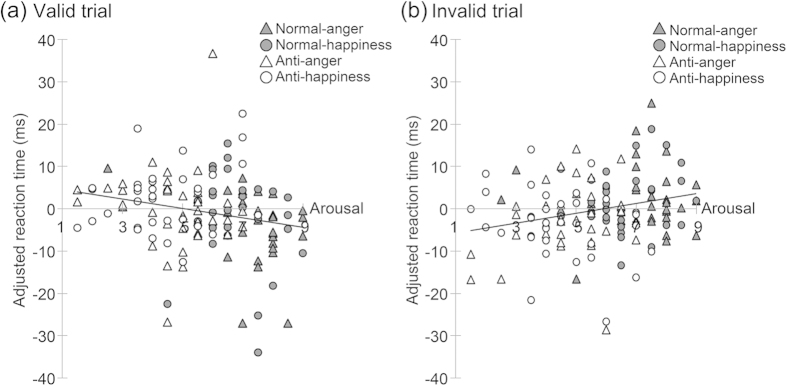
The relationship between arousal rating and RT in valid (a) and invalid trials (b). We calculated the adjusted RTs by partialling out the effects of participants to plot the relationship between arousal and RTs. The scatter plots and regression lines indicate the relationships between the arousal rating and adjusted RT.

All facial stimuli were chosen from the stimulus set of a previous study^9^. The schematic images of the facial stimuli were shown in Fig. 1(a), although actual stimuli were photographic faces. Normal expressions were grayscale photographs of a female (PF) and a male (PE) models with angry, happy, and neutral expressions that were drawn from a facial expression database^22^. Neither model was familiar to any of the participants. No expression showed bared teeth. Anti-expressions were created from the normal expressions using computer-morphing software (FUTON System, ATR-Promotions) by a previous study^9^. First, the researchers identified the coordinates of 79 facial feature points of normal expressions of emotional (anger and happiness) and neutral expressions manually and calculated the differences between the points of emotional and neutral facial expressions. Then, they determined the positions of the feature points for the anti-expressions by moving each point of neutral expressions by the same distance in the direction opposite from that in the normal emotional faces. Thus, the anti-expressions contained the same amount of visual changes as normal emotional expressions compared with neutral expressions. At last, all facial stimuli were cropped into an ellipse, and their contrasts were adjusted.

To confirm that stimulus faces could show expressions of the target emotions, we preliminarily conducted categorization task with 20 participants (10 females, *M* ± *SD* age, 28.9 ± 5.9 years), none of them participated in the cueing task. We showed each cue facial expression and asked participants to answer which of three labels (angry, happy, and neutral) described the facial expressions most appropriately. The results showed that normal-angry, normal-happiness, anti-angry, anti-happiness, and neutral expressions were most frequently categorized as angry (97.5%), happy (80.0%), neutral (72.5%), neutral (55.0%), and neutral (85.0%) expressions, respectively. These results replicated those in previous studies^8^,^9^ showing that anti-expressions depicted neutral emotions.

### Apparatus

The stimulus presentation was controlled by Presentation 14.9 (Neurobehavioral Systems) installed on a Windows computer (HP Z200 SFF, Hewlett-Packard Company). The stimuli were presented on a 19-inch CRT monitor (HM903D-A, iiyama) with a refresh rate of 150 Hz and resolution of 1024 × 768 pixels. Participants sat in chairs 50 cm from the monitor. They used a keyboard (KU-0316, Hewlett-Packard Company) for their responses of the task.

### Experimental procedure

Experiments were conducted individually in a quiet room. The participants engaged in the cueing task and then the rating task.

#### Cueing task

The task consisted of a total of 160 trials presented in four blocks of 40 trials. The number of trials for each cue stimulus was 32 trials. The number of both valid and invalid trials was 16 trials for each cue condition with an equal number of left and right presentations of cues. The trial order was randomized across all cue-stimulus conditions within a block. Before the task began, participants underwent 20 practice trials to become familiar with the procedure.

Each trial started with 500-ms presentation of a fixation cross subtending a visual angle of 0.9° × 0.9° in the center of the display. Then, the facial cue was presented for 100 ms in either the left or the right visual field (the inside edge was 6.5° peripheral from the center). Finally, after 50-ms delay, the dot target was presented at either the same (valid trial) or the opposite (invalid trial) location of the facial cue until a response was made (Fig. 1(b)). After the response, the screen went blank for an inter-trial interval, which varied randomly from 1100 to 1500 ms.

Participants were required to keep their gaze on the fixation cross while the cross was presented and to localize the open dot as quickly and accurately as possible, whether the target appeared on the left or the right side of the display, by pressing left and right control keys on a keyboard, using left or right index finger, respectively. They were told that the stimuli preceding the targets were not predictive of the target location.

#### Rating

After the cueing task, all facial stimuli were presented individually to the participants, who were asked to evaluate each stimulus in terms of emotional arousal and valence subjectively experienced (i.e., the intensity and quality of the emotion that participants felt when perceiving the stimulus facial expression) using a nine-point scale ranging from 1 (low arousal and negative valence) to 9 (high arousal and positive valence). We also asked participants to rate familiarity (i.e., the frequency with which they encountered the facial expressions depicted by the stimulus in daily life) and naturalness (i.e., the degree to which the stimulus expressions seemed natural) of stimuli as possible confounding factors^13^. The order of presentation of the stimuli and rating items was randomized and balanced across participants.

### Data analyses

All statistical tests were performed using the IBM SPSS Statistics 22 (IBM Corporation). Accuracy for target localization task was higher than 98.3% under all cue conditions. We found no evidence of a speed-accuracy tradeoff.

#### RT

The mean RTs for correct responses to the target localization task were calculated for valid and invalid trials under each facial cue condition, excluding measurements beyond the mean ±3 *SD*s as artifacts (*M* ± *SD*, 1.9 ± 1.7%). The RTs were subjected to a three-way repeated-measure ANOVA with type (normal/anti-expression) and emotion (anger/happiness), and validity (valid/invalid) as within-participant factors. Follow-up analyses of the simple-effect were conducted following significant interactions^23^. RTs were also subjected to multiple comparison analyses with Bonferroni correction to compare the normal/anti-expression condition with the neutral expression condition.

#### Rating

Each rating of arousal, valence, familiarity, and naturalness was subjected to a two-way repeated-measure ANOVA with type (normal/anti-expression) and emotion (anger/happiness). Follow-up analyses of the simple-effect were conducted following significant interactions^23^. Subjective ratings were also subjected to multiple comparison analyses with Bonferroni correction to compare the normal/anti-expression condition with the neutral expression condition.

#### Rating-RT relationship

We performed a multiple regression analysis for each validity condition using the mean RT for each participant under each cue condition as the dependent variable and the mean ratings (arousal, valence, familiarity, and naturalness) and dummy variables for participants as the independent variables. We calculated the adjusted RTs by partialling out the effects of participants to show the relationship between the rating and RTs.

## Additional Information

**How to cite this article**: Sawada, R. and Sato, W. Emotional attention capture by facial expressions. *Sci. Rep*. **5**, 14042; doi: 10.1038/srep14042 (2015).

## Figures and Tables

**Table 1:** Mean (with SE) subjective rating scores of arousal, valance, familiarity, and naturalness for normal and anti-expressions of anger and happiness and neutral expression.

Items	Normal		Anti		Neutral
	Anger	Happiness	Anger	Happiness	
Arousal	7.06 (0.27)	6.69 (0.20)	4.80 (0.25)	4.36 (0.25)	3.77 (0.22)
Valence	2.63 (0.18)	7.02 (0.24)	4.88 (0.20)	3.89 (0.18)	5.17 (0.12)
Familiarity	3.38 (0.26)	6.95 (0.22)	5.02 (0.21)	4.11 (0.22)	6.31 (0.23)
Naturalness	4.75 (0.33)	6.97 (0.27)	5.09 (0.30)	5.02 (0.32)	7.00 (0.26)
